# An ancient role for collier/Olf/Ebf (COE)-type transcription factors in axial motor neuron development

**DOI:** 10.1186/s13064-018-0125-6

**Published:** 2019-01-18

**Authors:** Catarina Catela, Edgar Correa, Kailong Wen, Jihad Aburas, Laura Croci, G. Giacomo Consalez, Paschalis Kratsios

**Affiliations:** 1 Department of Neurobiology, University of Chicago, Chicago, IL USA; 2 Division of Neuroscience, San Raffaele Scientific Institute, Milan, Italy; 3 Università Vita-Salute San Raffaele, Milan, Italy

## Abstract

**Background:**

Mammalian motor circuits display remarkable cellular diversity with hundreds of motor neuron (MN) subtypes innervating hundreds of different muscles. Extensive research on limb muscle-innervating MNs has begun to elucidate the genetic programs that control animal locomotion. In striking contrast, the molecular mechanisms underlying the development of axial muscle-innervating MNs, which control breathing and spinal alignment, are poorly studied.

**Methods:**

Our previous studies indicated that the function of the Collier/Olf/Ebf (COE) family of transcription factors (TFs) in axial MN development may be conserved from nematodes to simple chordates. Here, we examine the expression pattern of all four mouse COE family members (*mEbf1-mEbf4*) in spinal MNs and employ genetic approaches in both nematodes and mice to investigate their function in axial MN development.

**Results:**

We report that *mEbf1* and *mEbf2* are expressed in distinct MN clusters (termed “columns”) that innervate different axial muscles. Mouse *Ebf1* is expressed in MNs of the hypaxial motor column (HMC), which is necessary for breathing, while *mEbf2* is expressed in MNs of the medial motor column (MMC) that control spinal alignment. Our characterization of *Ebf2* knock-out mice uncovered a requirement for *Ebf2* in the differentiation program of a subset of MMC MNs and revealed for the first time molecular diversity within MMC neurons. Intriguingly, transgenic expression of *mEbf1* or *mEbf2* can rescue axial MN differentiation and locomotory defects in nematodes (*Caenorhabditis elegans*) lacking *unc-3*, the sole *C. elegans* ortholog of the COE family, suggesting functional conservation among *mEbf1, mEbf2* and nematode UNC-3.

**Conclusions:**

These findings support the hypothesis that genetic programs controlling axial MN development are deeply conserved across species, and further advance our understanding of such programs by revealing an essential role for *Ebf2* in mouse axial MNs. Because human mutations in COE orthologs lead to neurodevelopmental disorders characterized by motor developmental delay, our findings may advance our understanding of these human conditions.

**Electronic supplementary material:**

The online version of this article (10.1186/s13064-018-0125-6) contains supplementary material, which is available to authorized users.

## Background

**Fig. 1:**
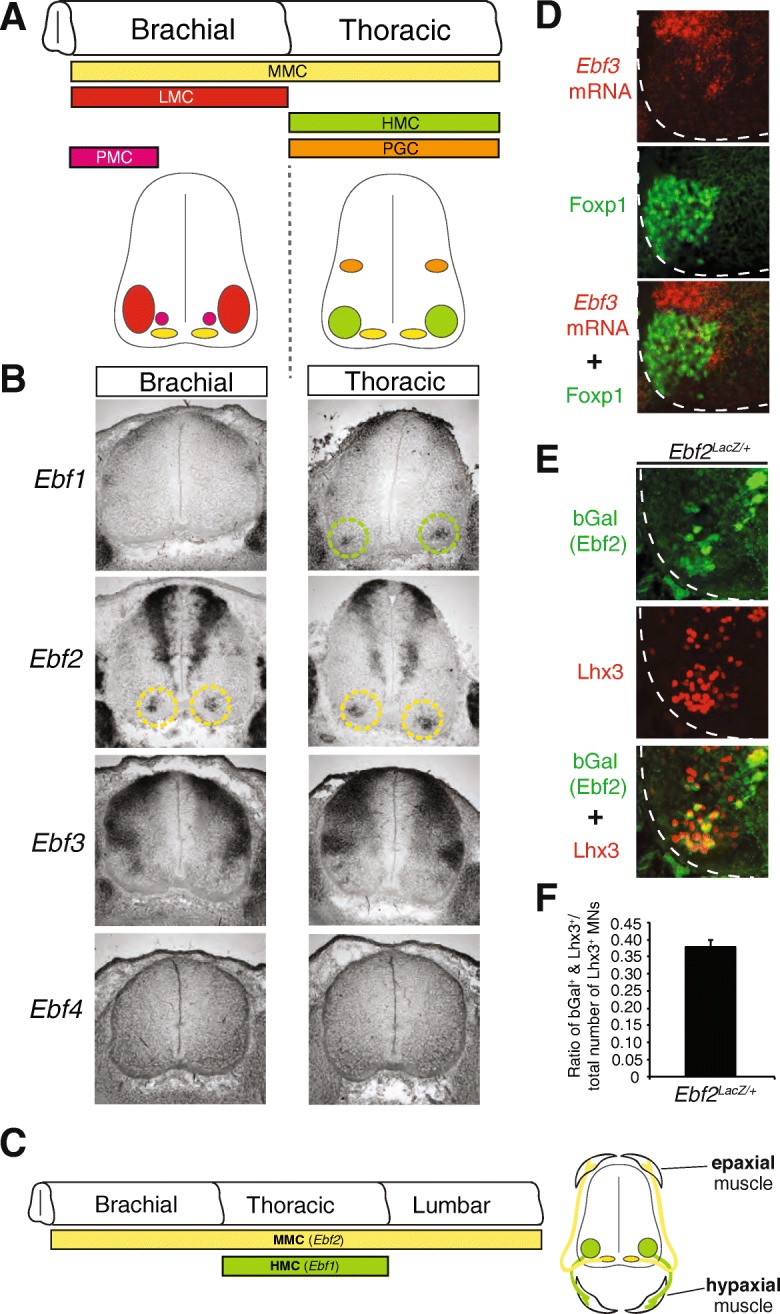
Ebf1 and Ebf2 are expressed in axial muscle-innervating motor neurons. **a** Schematic of the spinal cord showing different columns of MNs (color-coded) at distinct regions along the rostro-caudal axis (brachial and thoracic). A cross-section of each region is provided below. **b** RNA ISH analysis for mouse *Ebf1-Ebf4* at e13.5 of WT spinal cords. **c** Schematic summarizing the expression of *Ebf1* (HMC) and *Ebf2* (MMC) based on data from panel B. On the right, axonal projections are schematized of HMC and MMC neurons to hypaxial and epaxial muscles, respectively. **d** Antibody staining for the LMC marker (Foxp1, green signal) combined with fluorescent RNA ISH for *Ebf3* (red signal) revealed almost no co-localization in WT e13.5 spinal cord. *N* = 3. **e-f**: Double immunostaining for Lhx3 (MMC marker in red) and bGal (*Ebf2* reporter in green) shows that ~ 40% of MMC neurons express *Ebf2*. Quantification (**f**) provided as the ratio of MNs that are double positive for Lhx3 and bGal over the total number of Lhx3 positive MNs. For this analysis, *Ebf2* ^*LacZ/+*^ embryos were used at e12.5. *N* = 5

The mammalian neuromuscular system is essential for distinct motor behaviors ranging from locomotion and dexterity to basic motor functions, such as breathing and maintenance of spinal alignment [1]. The underlying basis for achieving these diverse outputs lies in the assembly of distinct neuronal circuits dedicated to control different muscles. In the mouse spinal cord, for example, these circuits are composed of various motor neuron (MN) subtypes organized into distinct clusters of cells (termed “columns”) along the rostrocaudal axis (Fig. 1a). At the brachial and lumbar levels, MNs of the lateral motor column (LMC) innervate limb muscles, which are essential for locomotion and dexterity [2, 3]. Breathing is controlled by cervical MNs of the phrenic motor column (PMC) that innervate the diaphragm, and by thoracic MNs of the hypaxial motor column (HMC) that innervate hypaxial (intercostal and abdominal) muscles (Fig. 1c). In contrast to these segmentally-restricted columns (LMC, PMC, HMC), MNs of the medial motor column (MMC) are generated along the entire length of the spinal cord and innervate epaxial (back) muscles necessary for maintenance of spinal alignment [1]. (Fig. 1a, c). In recent years, remarkable progress has been made in deciphering the molecular mechanisms that specify limb-innervating MNs (LMC). However, the genetic programs underlying the development of hypaxial (HMC) and epaxial (MMC) muscle-innervating MNs, which control more than half of all skeletal muscles in mammals, are poorly understood [1].

The identity of MN subtypes that belong to a specific column, termed “columnar identity”, is defined by combinatorial expression of transiently expressed TFs [2, 4, 5]. For example, LMC columnar identity is imposed by co-expression of several LIM homeodomain proteins (Islet1, Islet2, Hb9 [Mnx1]) and the Forkhead box protein P1 (Foxp1). On the other hand, combinatorial expression of Islet1, Islet2, Lhx3 and Hb9 demarcates MMC columnar identity [6]. The highly conserved Hox proteins also play key roles in establishing columnar MN identity. Distinct combinations of Hox genes are expressed in MNs along the rostrocaudal axis of the spinal cord [3, 7]. At brachial and lumbar levels, for example, Hox genes control LMC identity through the direct activation of Foxp1 [8]. However, the identity of the non-segmentally restricted MMC neurons appears to be insensitive to the activities of Hox proteins [1, 9], suggesting that distinct genetic programs have evolved for the establishment of limb-innervating (LMC) and axial muscle-innervating (MMC) MNs.

A large body of work on limb-innervating MNs (LMC) demonstrated that combinatorial TF expression is also necessary for further diversification of MNs within the same column, providing evidence for molecular diversity within the LMC column. This molecular diversity of LMC MNs correlates with innervation of distinct muscle groups [4, 5, 10]. For example, in the mouse hind limb, co-expression of Islet1, Islet2, Er81 and Nkx6.1 is necessary for specification of MNs located in medial LMC (LMCm) that innervate ventral limb muscles, whereas a different combination of TFs defines lateral LMC (LMCl) identity, thereby ensuring proper innervation of dorsal limb muscles. Unlike LMC MNs, it remains unclear to date whether molecular diversity exists within the axial muscle-innervating MNs. While combinatorial expression of Islet1, Islet2, Lhx3 and Hb9 demarcates MMC columnar identity [1, 6], molecular markers that label subsets of MMC neurons have not been identified in mice. Since MMC neurons innervate different types of epaxial muscle, such as spinalis, longissimus, and iliocostalis [11], is this anatomical diversity accompanied by molecular diversity?

MMC neurons are thought to represent the ancestral “ground state” from which all other spinal cord MN subtypes subsequently evolved [2]. This notion is supported by the fact that “MMC-like” neurons innervate axial/body-wall muscles in limbless vertebrates (e.g., lamprey), insect larvae (e.g., *Drosophila melanogaster*) and nematodes (e.g., *Caenorhabditis elegans*), suggesting that an MMC-like population likely represents the ancestral condition of MNs in bilaterians [1]. It is therefore not surprising that at least some of the intrinsic genetic programs of axial MN development appear to be conserved from invertebrates to vertebrates. Supporting this notion, “MMC-like” neurons in *Drosophila* and *C. elegans* can be defined, similar to vertebrates, by the expression of Hb9, Lhx3 and Islet1/2 orthologs [12]. Cross-species comparisons have also revealed “lost” mechanisms. This is perhaps best exemplified by the case of the conserved homeodomain TF *even-skipped (eve)*. Drosophila eve and its *C. elegans* ortholog *vab-7* are utilized for specification of body wall muscle-innervating MNs [13–17], while the *eve/vab-7* mouse ortholog Evx1 is not involved in MN specification. Instead, Evx1 is strictly required for V0 spinal interneuron fate [18]. Although axial MNs are used for distinct motor functions in different species (locomotion in limbless vertebrates, insect larvae and nematodes versus maintenance of spinal alignment in mammals), the aforementioned examples collectively illustrate that cross-species comparisons can reveal the extent of conservation in the genetic programs underlying axial MN development.

Our previous studies in the nematode *C. elegans* revealed that UNC-3, the sole ortholog of the Collier/Olf/Ebf (COE) family of TFs, is required for differentiation of body wall muscle-innervating MNs that control locomotion [19–21]. COE family orthologs are expressed in the nervous system of very distant species ranging from cnidarians (e.g., sea anemone) [22] to bilaterians (nematodes [23–25], annelids [26], flies [27], frogs [28], zebrafish [29], mice [30–36]), indicating an ancient role for COE factors in nervous system development. Functional studies have shown that the sole *Drosophila* COE ortholog *collier (or knot)* is required for peptidergic neuron specification [37–40], and COE orthologs in frog and chick embryos function to promote neuronal differentiation [28, 41]. Four COE orthologs are embedded in the mouse genome, mEbf1-mEbf4. Previous reports have identified mEbf1 as a key player in facial MN migration, as well as neuronal differentiation in the striatum and retina [32, 33, 42]. Mouse Ebf2 is required for neuronal cell migration and differentiation in the cerebellum and olfactory epithelium, where it is believed to function in a partially redundant manner with Ebf3 [30, 31, 36, 43, 44]. However, the function of mouse Ebf proteins in spinal MN development remains poorly understood.

Our previous studies on cholinergic MNs of *C. elegans* and the simple chordate *Ciona intestinalis* indicated that the function of UNC-3 is conserved from nematodes to simple chordates [21]. Here, we provide evidence that the function of UNC-3 may be conserved from *C. elegans* to mammals. We found, in mice, that mEbf1 and mEbf2 are implicated in axial MN development. In the embryonic mouse spinal cord, mEbf1 is selectively expressed in hypaxial muscle-innervating MNs (HMC), while mEbf2 is expressed in ~ 40% of epaxial muscle-innervating MNs (MMC), providing the first evidence for molecular diversity within MMC neurons in mice. Using Ebf2 KO mice, we assessed in vivo the function of Ebf2 and revealed its requirement for differentiation of a subset of MMC neurons. Lastly, cross-species transgenic rescue experiments demonstrated that mouse Ebf1 or Ebf2 can functionally substitute for nematode UNC-3. Altogether, our study uncovers an ancient role for COE family TFs in axial MN development. Because human mutations in COE orthologs cause neurodevelopmental disorders characterized by motor developmental delay [45–49], our findings could help advance our understanding of those conditions.

## Methods

### Mouse husbandry

All mouse procedures were approved by the Institutional Animal Care and Use Committee (IACUC) of the University of Chicago.

### *RNA* in situ *hybridization,* antibody staining and quantification

**Fig. 2:**
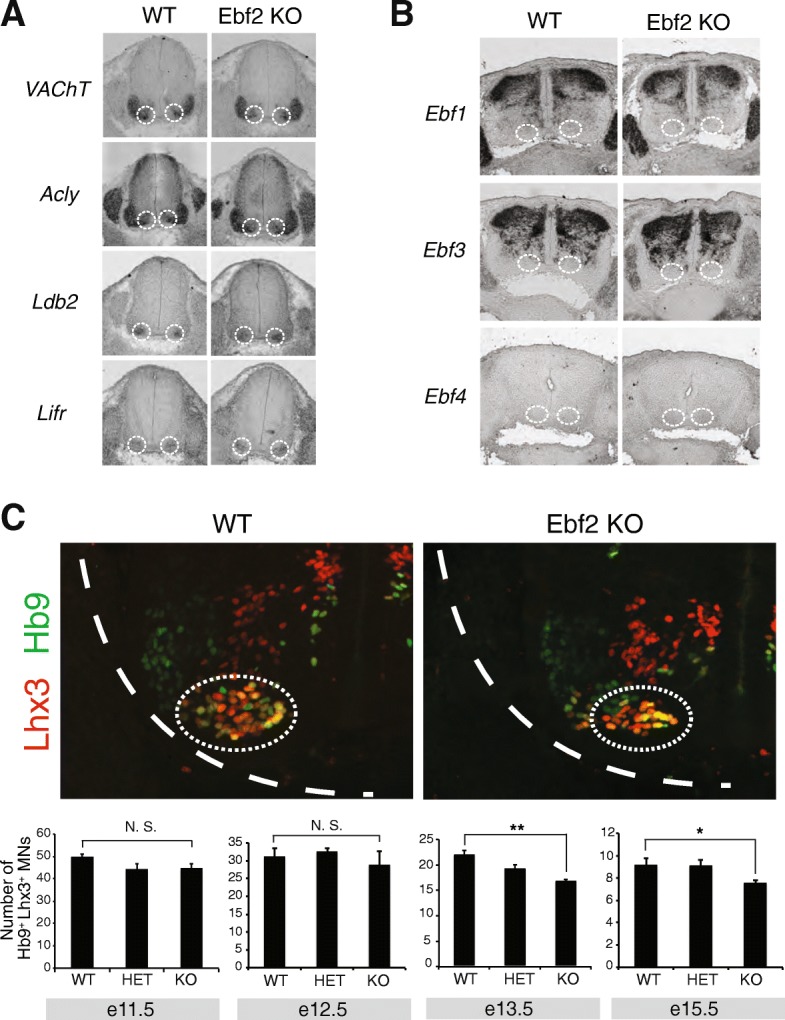
Characterization of MMC neurons in Ebf2 KO animals. **a** The expression of genes involved in ACh biosynthesis (VAChT, Acly), as well as the expression of the MMC-specific genes (*Ldb2, Lifr*) is not affected in MMC neurons (white dotted circles) of Ebf2-KO spinal cords at e13.5. *N* = 3. **b** RNA ISH analysis on Ebf2-KO spinal cords at e12.5 reveals no compensatory up-regulation of *Ebf1, Ebf3* and *Ebf4* transcripts in MMC neurons (white dotted circles). Similar results were obtained at e13.5. *N* = 3. **c** Two representative images are shown from e13.5 WT and Ebf2 KO spinal cords. The number of MMC neurons co-labeled with Lhx3 (red) and Hb9 (green) is not affected in Ebf2 KO spinal cords at e11.5 and e12.5, but is significantly decreased at e13.5 and e15.5. Student’s t-test was performed. *: *p* < 0.05, **: *p* < 0.01. *N* = 4–7. The location of MMC neurons is indicated with a dotted white circle

**Fig. 3:**
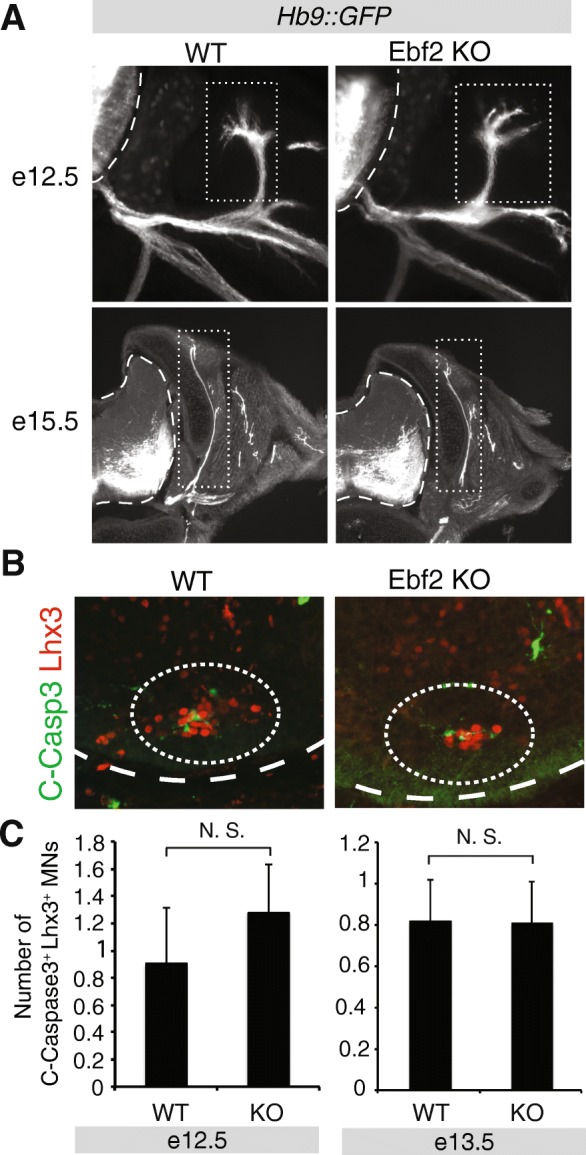
No evidence for axon guidance defects and cleaved Caspase 3-mediated cell death in MMC neurons of Ebf2 KO mice. **a** Motor neuron axons (bundled into nerves) are visualized with the Hb9::GFP transgene in WT and Ebf2 KO spinal cords at e12.5 and e15.5. The MN axons that belong to MMC (dorsal nerve) are indicated with a white dotted box. Hb9::GFP signal is shown in white for better contrast. *N* = 4. The spinal cord boundary is indicated with a white dotted line. **b** Representative images of WT and Ebf2 KO spinal cords (cross-sections) immunostained with antibodies against Cleaved-Caspase 3 (C-Casp3 in green) and Lhx3 (MMC marker in red) at e12.5. The location of MMC neurons is circled. **c** Quantification of the number of MMC neurons (Lhx3^+^) that are also positive for C-Casp3 staining in WT and Ebf2 KO spinal cords. This analysis was performed at e12.5 and e13.5. N. S. indicates no significant differences were found with Student’s t-test. *N* = 4

Embryos were harvested at e12.5, e13.5, and e15.5, fixed in 4% paraformaldehyde for 1.5–2 h, placed in 30% sucrose over-night (4 °C), and embedded in optimal cutting temperature (OCT) compound. Cryosections were generated and processed for in situ hybridization or immunohistochemistry using antibodies against Foxp1, Lhx3, Hb9, and bGal as previously described [10, 50]. Images were obtained with a high-power fluorescent microscope (Zeiss Imager.V2) and analyzed with Fiji software [51]. Cell counting was performed using ImageJ software in at least three embryos per genotype. Cells stained for Foxp1, Lhx3, Hb9, bGal (Ebf2) were counted at the same position along the spinal cord for each genotype using the Cell count plug-in. We note that Lhx3 is expressed in both MMC neurons and v2a interneurons. In Fig. 2, we identified the MMC neurons by co-expression of Lhx3 and Hb9. In Figs. 1 and 3, we identified the MMC neurons based on Lhx3 expression and cell body position (MMC neurons are more ventrally located compared to v2a neurons). The axial projections of Hb9-GFP labeled MNs were visualized in vibratome sections (150 μm) of control (*Hb9-GFP*) and *Ebf2 KO; Hb9-GFP* spinal cords at e12.5 and e15.5.

### Analysis of somatotopic organization of Ebf2-expressing MMC neurons

The distribution of Ebf2 positive motor neurons along the medio-lateral axis of the MMC at e12.5 was analyzed with the Image J plot profile plugin. Following antibody staining for bGal (*Ebf2* reporter) on Ebf2^lacZ/+^ embryos, bGal fluorescence intensity was plotted along the medio-lateral region demarcated by MMC (Lhx3+) cells. Three distinct locations along the spinal cord were analyzed (rostral brachial, caudal brachial, thoracic) in 3 e12.5 embryos for each genotype.

### *C. elegans* strains

Nematode *C. elegans* strains were maintained as previously described [52]. Two strong loss-of-function (null) alleles (*e151, n3435*) for *unc-3* were used in this study [24]. Cholinergic MN reporters used: *juIs14 [acr-2*^*prom*^*::gfp], vsIs48 [unc-17*^*prom*^*::gfp]*. The following rescuing transgenic strains using the MN-specific promoter of *unc-3*^*prom*^ (558 bp) were generated: two rescuing transgenic lines (*a: kasEx95, b: kasEx96*) for *MN > mEbf1 in the unc-3 (n3435); juIs14 background,* two rescuing transgenic lines (*a: kasEx97, b: kasEx98*) for *MN > mEbf2 in the unc-3 (n3435); juIs14* background, two rescuing transgenic lines (*a: kasEx99, b: kasEx100*) for *MN > mEbf1 in the unc-3 (e151); vsIs48* background, and two rescuing transgenic lines (*a: kasEx101, b: kasEx102*) for *MN > mEbf2 in the unc-3 (e151); vsIs48* background.

### Rescue experiments in *C. elegans*

The *unc-3*^*prom*^*::mEbf1::unc-54 3’UTR* construct and the *unc-3*^*prom*^*::mEbf2::unc-54 3’UTR* construct were generated to contain a 558 bp proximal fragment (immediately upstream of ATG) of the *unc-3* promoter amplified from *C. elegans* wild type (N2) genomic DNA. In addition, each construct contains corresponding *mEbf1* or *mEbf2* coding sequences amplified from mouse cDNA and fused to *C. elegans unc-54* 3’UTR. Fragments were assembled through the Gibson Assembly method, which can be found at https://www.neb.com/applications/cloning-and-synthetic-biology/dna-assembly-and-cloning/gibson-assembly. These constructs were injected at 25 ng/μl into both *unc-3(n3435); juIs14 [acr-2*^*prom*^*::gfp] and unc-3(e151); vsIs48 [unc-17*^*prom*^*::gfp]* mutant strains together with *myo-2*^*prom*^*::GFP* (co-injection marker results in *gfp* labeled pharynx) and a carrier plasmid that serves as filler DNA (pBluescript). At least two stable transgenic lines were established for each construct and tested for rescue of the *unc-3* mutant phenotypes.

### Body bend/thrashing assay

Young-adult nematodes (2 days old) were transferred to an NGM plate containing a droplet (100 μl) of M9 buffer. After 1 min of adaptation, the number of body bends for 30 s was quantified as previously described [53]. A movement of the worm that swings its head and/or tail to the same side is counted as one thrash.

### Phylogenetic tree for COE family members

**Fig. 4:**
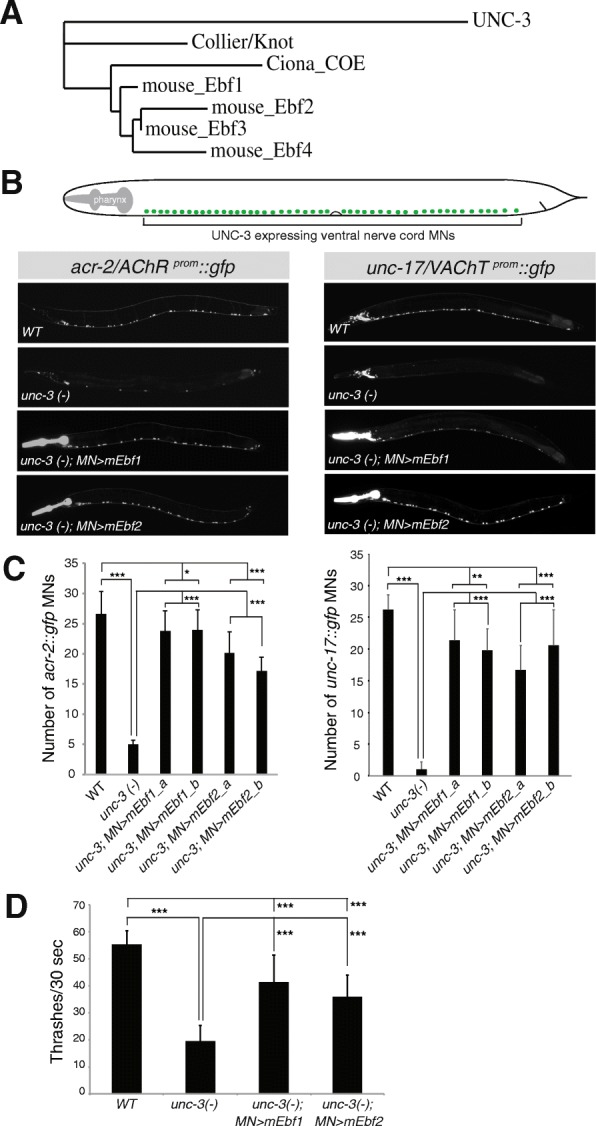
Mouse Ebf1 and Ebf2 rescue MN differentiation and locomotory defects in nematodes lacking *unc-3*. **a** Phylogenetic tree generated using Phylogeny.fr [54–56]. **b** Schematic showing the cell body position of ventral nerve cord MNs (labeled with green) in *C. elegans.* Forty [40] of these MNs are cholinergic and express UNC-3. The expression of two cholinergic MN markers (*acr-2*/AChR, *unc-17*/VAChT) is dramatically reduced in *unc-3* mutant animals carrying loss-of-function alleles (*e151, n3435*). Transgenic expression of mEbf1 *[Punc-3::mEbf1::unc-54 3’ UTR]* or mEbf2 *[Punc-3::mEbf2::unc-54 3’ UTR]* significantly restores expression of *acr-2*/AChR and *unc-17*/VAChT in MNs of *unc-3* mutants. Two transgenic lines per construct were used. A representative image of one rescuing line is shown. The pharynx is *gfp* positive because a pharyngeal promoter (*myo-2*) fused to *gfp* was used as a co-injection marker to distinguish transgenic from non-transgenic worms (see Methods). Fifteen animals at larval stage 4 (L4) were imaged for each genotype. Note that the fragment of *unc-3* promoter (558 bp) used for rescue drives expression of mEbf1 or Ebf2 in ~ 30 MNs of the *C. elegans* ventral nerve cord. Expression of *acr-2*/AChR or *unc-17*/VAChT in these 30 MNs is significantly affected in *unc-3 (−)* mutant nematodes, and partially restored when either mEbf1 or mEbf2 is provided using the *unc-3*558 bp MN-specific promoter. **c** Quantification of the results shown in panel B. Two transgenic lines (indicated with a and b) per rescuing construct were quantified. Student’s t-test was performed. *N* = 15. *: *p* < 0.05, **: *p* < 0.01, ***: *p* < 0.001. Statistical comparison between WT and MN > mEbf1/2 lines indicates partial rescue. **d** Thrashing assay shows that the movement defects of *unc-3* mutant nematodes can be significantly restored upon MN-specific expression of mEbf1 or mEbf2. Statistical comparison between WT and MN > mEbf1/2 lines indicates partial rescue. Two day-old adult nematodes were used. *N* = 10. Student’s t-test was performed. *: *p* < 0.05, **: *p* < 0.01, ***: *p* < 0.001

The peptide sequence for UNC-3 and Collier (Knot) were recovered from WormBase and FlyBase, respectively. The peptide sequences for mEbf1, mEbf2, mEbf3, mEbf4, and *Ciona intestinalis* COE were downloaded from Ensembl Genome Browser. While a single isoform was found for UNC-3, Collier and *Ciona* COE, several protein isoforms for mouse Ebfs were reported in Ensembl. The longest mEbf1, mEbf2, mEbf3, and mEbf4 isoforms were considered for the generation of the phylogenetic tree shown in Fig. 4a. Phylogenetic analysis was performed at http://phylogeny.lirmm.fr using the “one-click” mode [54–56].

### Statistical analysis

For data quantification, graphs show values expressed as mean ± standard deviation (SD). Statistical significance was determined with the unpaired two-tailed Student’s t test using Microsoft Excel.

## Results

### Ebf1 and Ebf2 are expressed in distinct MN populations that innervate axial muscles

Since orthologs of COE family TFs control aspects of MN differentiation in the nematode *C. elegans* and the simple chordate *C. intestinalis* [21], we hypothesized that the function of COE TFs is conserved in mouse MNs. Four orthologs of the COE family are embedded in the mouse genome (mEbf1 - mEbf4). We therefore sought to examine the expression pattern of all four mEbfs in the developing mouse spinal cord through RNA in situ hybridization (ISH). Since different motor columns are found along the rostro-caudal axis of the spinal cord (Fig. 1a), we performed RNA ISH for mEbf1–4 at brachial, thoracic, and lumbar levels. We chose embryonic day 13 (e13.5) as the most appropriate time point for this analysis because post-mitotic MNs by e13.5 are organized into columns that can be distinguished with molecular markers (Fig. 1a). We found that mEbf1 is expressed in MNs of the HMC but no other column at brachial, thoracic, or lumbar levels, constituting a highly specific, post-mitotic marker for HMC neurons (Fig. 1b). This finding was further corroborated by coupling of Ebf1 RNA ISH with antibody staining for the MN marker Hb9 (Additional file 2: Figure S1C). Unlike mEbf1, mEbf2 is selectively expressed in MNs along the entire length of the spinal cord that belong to MMC (Fig. 1b, c), a finding we confirmed using additional criteria, such as cell body position along the dorso-vental axis and a molecular marker (Lhx3) (see next section). Our RNA ISH revealed expression of mEbf3 in the ventral zone of e13.5 spinal cords, a territory close to the location of LMC MNs. However, coupling of mEbf3 RNA ISH analysis with antibody staining for Foxp1, a LMC-specific marker, revealed almost no co-localization (Fig. 1d). Occasionally, we found 1 or 2 Foxp1+ nuclei (in green) that co-localized with *Ebf3* mRNA signal (in red). We also detected mEbf3 expression in interneurons of the intermediate and dorsal zones, as well as dorsal root ganglia (DRG) (Fig. 1b**,** Additional file 2: Figure S1A). We note that mEbf1 and mEbf2 expression was also detected in dorsal interneurons and DRG neurons (Fig. 1b**,** Additional file 2: Figure S1A). No expression for mEbf4 was detected in spinal cord and DRG neurons at e13.5 (Fig. 1b, Additional file 2: Figure S1). These findings extend previous observations of the Ebf1–3 expression pattern in the spinal cord performed at earlier stages, before MNs are organized into columns [33]. By examining the expression pattern of all members of the COE family of TFs at e13.5 spinal cords, we demonstrate that mEbf1 and mEbf2 are selectively expressed in axial muscle-innervating MNs of the HMC and MMC, respectively. Based on molecular and positional criteria (Fig. 1**,** Additional file 2: Figure S1), we found no evidence for Ebf1 and Ebf2 expression at e13.5 in the remaining motor columns, such as the limb-innervating MNs of the LMC.

### mEbf2 is required for differentiation of a subset of MMC neurons

The mEbf2 expression in MNs along the spinal cord (MMC) is reminiscent of the UNC-3 expression pattern in body wall muscle-innervating MNs along the *C. elegans* nerve cord [21, 25]. We therefore hypothesized that – similar to its *C. elegans* ortholog (UNC-3) – mEbf2 is also required for differentiation of mouse MNs (MMC) that innervate body wall muscles, and more precisely epaxial muscles. Before testing this hypothesis, it is necessary to determine whether all MMC neurons or a fraction of them express mEbf2 (Fig. 1b). To this end, we used a mouse line, which carries a promoterless lacZ cDNA cassette (without a nuclear localization signal [NLS]) to replace the first five exons of the endogenous Ebf2 locus [43]. Heterozygous Ebf2 ^LacZ/+^ mice enabled us to perform double immunofluorescence staining for b-galactosidase (produced by Lacz), which serves as an Ebf2 reporter, and the molecular marker Lhx3 that specifically labels MMC neurons in the ventral-most part of the spinal cord [6] (see Methods). Following quantification of the number of MNs that co-express b-galactosidase (Ebf2) and Lhx3 at e13.5, we found that ~ 40% of the MMC neurons express Ebf2 at any given region (brachial, thoracic, or lumbar) of the spinal cord (Fig. 1e-f**,** Additional file 2: Figure S1B). This finding corroborates our RNA ISH analysis for mEbf2 expression in MMC (Fig. 1b), and further suggests that molecular differences do exist within MMC neurons.

Next, we aimed to identify which subpopulations of MMC neurons express Ebf2. Due to the lack of available molecular markers for subtypes of MNs within the MMC column of the mouse spinal cord, we first examined whether there is any somatotopic organization to the Ebf2-expressing MMC neurons. To this end, we plotted the distribution of Ebf2 positive neurons along the medio-lateral axis of the MMC at e12.5 (Additional file 1: Figure S2). By analyzing three distinct regions along the rostro-caudal axis of e12.5 spinal cords (rostral brachial, caudal brachial, thoracic) of Ebf2 ^LacZ/+^ mice, we detected a mild region-specific bias in the position of Ebf2 positive motor neurons. At rostral brachial and thoracic regions, Ebf2-expressing cells are mainly located at the center of the MMC column, whereas, at caudal brachial regions, Ebf2-expressing cells are mainly located more medially within the MMC (Additional file 1: Figure S2). Next, we took advantage of a previous study describing the organization and muscle connectivity of MNs in the thoracic segment of the rat spinal cord [57]. Based on that study and the cell body position of Ebf2 neurons (at the center of MMC) in the mouse thoracic spinal cord (Additional file 1: Figure S2), it is likely that the Ebf2-expressing MNs innervate the longissimus, iliocostalis or levator costae epaxial muscles.

To examine the function of Ebf2, we used Ebf2 ^LacZ/LacZ^ homozygous mice. These mice globally inactivate Ebf2 gene activity [43], and will be referred to as Ebf2 KO mice hereafter. Since orthologs of Ebf2, in nematodes and simple chordates, are required for expression of the highly conserved genes involved in acetylcholine (ACh) biosynthesis, we evaluated the expression of the ACh pathway genes VAChT and Acly by RNA ISH in MMC neurons of Ebf2 KO embryonic spinal cords (Fig. 2a). We did not detect any differences, a finding that raises the possibility of genetic redundancy among the 4 mEbf factors. However, RNA ISH analysis did not show any compensatory expression of Ebf1, Ebf3, and Ebf4 in MMC neurons of Ebf2 KO embryos (Fig. 2b). We therefore conclude that – unlike its nematode and simple chordate orthologs – Ebf2, in mice, does not disrupt the expression ACh pathway genes in body wall muscle-innervating MNs (MMC).

The expression of genes involved in ACh biosynthesis is a common feature shared by all spinal MNs irrespective of columnar identity. We then asked whether molecular features specific to MNs of the MMC column are affected in Ebf2 KO embryos. It is well established that co-expression of the TFs Lhx3 and Hb9 demarcates the MMC neurons in the developing spinal cord [6, 58]. We quantified the number of double-positive (Lhx3+ and Hb9+) MNs in the ventral zone of WT, Ebf2 heterozygous (Ebf2 ^LacZ/+^) and homozygous KO (Ebf2 ^LacZ/LacZ^) spinal cords at e11.5, e12.5, e13.5, and e15.5 (Fig. 2c). Although no differences were observed at e11.5 and e12.5, we found a statistically significant decrease in the number of MMC MNs (Lhx3+ and HB9+) in Ebf2 KO spinal cords at e13.5 and e15.5, suggesting that MMC neurons are normally generated in these mutants but Ebf2 is selectively required for later stages of MMC neuron differentiation (Fig. 2c). This possibility is further supported by the fact that Ebf2 expression in MMC is maintained throughout embryonic development (Additional file 2: Figure S1B). However, the expression of *Ldb2* and *Lifr*, two recently described molecular markers for MMC fate [9], appears unaffected (Fig. 2a). Lastly, we asked whether these molecular defects, i.e., decreased number of MMC MNs expressing Lhx3 and HB9 (Fig. 2c), are accompanied by anatomical defects in MMC neurons, such as inability to reach their epaxial muscle targets. To this end, we visualized all spinal MN axons (including the MMC axons) using the Hb9-GFP mouse line [59], which we crossed to Ebf2 KO mice. We were unable to detect any differences in the morphology or thickness of the MMC axonal bundle (nerve) that reaches epaxial muscles at e12.5 and e15.5 stages (Fig. 3a). We note, however, that the Hb9-GFP mouse line labels with GFP all MMC neurons, including the 40% of them that express Ebf2. This lack of specificity could contribute to our inability to detect any subtle MMC axonal defects in Ebf2 KO mice. On the other hand, MN axon guidance occurs earlier than e13.5, which is the earliest time point we detected a molecular defect in MMC neurons (Fig. 2c), indicating that Ebf2 may not control the early steps of MMC neuron differentiation. Altogether, our analysis suggests that Ebf2 is required for late differentiation of a subset of MMC neurons.

### The MMC differentiation defect in Ebf2 KO animals is not a result of Caspase 3-mediated cell death

The reduction in the number of MMC neurons co-expressing the molecular markers Lhx3 and Hb9 in Ebf2 KO embryos could be attributed either to a bona fide differentiation defect (i.e., MMC neurons are normally generated in the absence of Ebf2 gene activity, but fail to express Lhx3 and Hb9), or to a developmental defect that affects the number of MMC neurons (e.g., loss of Ebf2 triggers apoptosis in MMC neurons, thereby reducing their number). To investigate the latter possibility, we quantified the number of MMC neurons that express cleaved caspase-3 (C-Casp3), a well-documented pro-apoptotic marker and the converging point of several pro-apoptotic signaling pathways [60]. Upon double staining for Lhx3 (MMC marker) and C-Casp3, our quantification analysis at e12.5 and e13.5 revealed no statistically significant differences between WT (Ebf2 ^+/+^) and Ebf2 KO embryos (Fig. 3b-c). Although there is a possibility that we missed a narrow time window (between e12.5 and e13.5) during which increased apoptosis occurs, our C-Casp3 data thus far suggest that the MMC differentiation defect in Ebf2 mutant mice is not due to C-Casp3-dependent cell death that could affect the generation of MMC neurons. We note that this is also the case for nematodes lacking the Ebf2 homolog *unc-3*; axial MNs are normally generated in *unc-3* mutant animals but fail to express molecular markers for MN differentiation [21].

### Mouse Ebf1 and Ebf2 rescue MN differentiation defects in *unc-3* mutant nematodes

Our previous studies in the nematode *C. elegans* and the chordate *Ciona intestinalis* suggested that the function of UNC-3 in controlling axial MN differentiation is conserved from nematodes to simple chordates [21]. Here, we investigate whether the function of UNC-3 is conserved from nematodes to mammals through cross-species rescue experiments. Given that mEbf1 and mEbf2 are expressed in mouse axial MNs (Fig. 1a-c) and are phylogenetically closer to UNC-3 (based on sequence similarity) than mEbf3 and mEbf4 (Fig. 4a**)**, we sought to explore the functional conservation of mEbf1 and mEbf2 and nematode UNC­3. To this end, we expressed the mEbf1 or mEbf2 coding sequence specifically in MNs of *unc-3* mutant nematodes that carry strong loss-of-function or null *unc-3* alleles (*e151, n3435*) [24]. As assessed by analyzing the expression of two *unc-3*-dependent MN differentiation markers (*acr-2: ACh receptor subunit 2; unc-17: vesicular ACh transporter [VAChT]* [61]) [22], we found that both mEbf1 and mEbf2 could significantly rescue the *unc-3* mutant phenotype and restore *acr-2* and *unc-17* expression, albeit not to WT levels (Fig. 4b-c). In addition to evaluating expression of MN-specific molecular markers, we asked whether MN-specific expression of mEbf1 or mEbf2 can rescue the severe locomotory defects previously observed in *unc-3* null nematodes [52]. To test this possibility, we employed a well-established thrashing assay [53] and counted the number of body bends per 30 s when adult worms are placed into a buffer solution (Fig. 4d). Our quantification revealed that MN-specific expression of either mEbf1 or mEbf2 in *unc-3* null animals resulted in a statistically significant increase in the number of body bends/30s when compared to *unc-3* null animals that do not carry the mEbf1 or mEbf2 rescuing transgenes, indicating partial rescue of the *unc-3* locomotory defects (Fig. 4d). Apart from corroborating the cell autonomy of the *unc-3* mutant phenotype (because mEbf1 and mEbf2 were expressed under the control of a MN-specific promoter [*unc-3* ^*558bp prom*^]), our behavioral and molecular analysis (*acr-2, unc-17*) show that mEbf1 and mEbf2 can functionally compensate for the lack of nematode *unc-3*.

## Discussion

While there is a remarkable wealth of developmental studies focusing on limb-muscle innervating MNs, the development of axial muscle-innervating MNs, which control more than half of all skeletal muscles in mammals, has received much less attention [1]. In this paper, we provide evidence for an ancient role of the COE family of TFs in axial MN development. First, we find that mEbf1 is a novel post-mitotic marker for HMC neurons, which innervate hypaxial muscles necessary for breathing. This finding paves the way for future studies aiming to illuminate the poorly explored genetic programs of HMC specification. Second, this study advances our understanding of the genetic programs underlying axial MN development: (**i**) By uncovering an essential role for mEbf2 in the differentiation of MMC neurons, which innervate epaxial muscle and control spinal alignment, and (**ii**) By revealing that molecular diversity does exist within MMC neurons as Ebf2 is expressed in a significant fraction (~ 40%) of these MNs. Third, our cross-species transgenic rescue experiments provide evidence for functional equivalence between COE orthologs, suggesting that the function of these TFs in the development of axial muscle-innervating MNs is conserved from nematodes to mammals. Altogether, this study indicates that a viable approach for advancing our understanding of axial MN development may lie in comparative studies of the genetic programs that specify invertebrate and vertebrate MNs.

The requirement of Ebf2 for differentiation of a subset of MMC neurons is reminiscent of previous observations in the cerebellar cortex [31, 62], where Ebf2 is selectively required for the differentiation of a subset of Purkinje neurons, indicating that Ebf2 may be important for diversification of highly similar neurons, such as MMC or Purkinje neurons, in the mammalian nervous system. A current limitation in our Ebf2 KO analysis is the lack of additional molecular markers for MMC neuronal differentiation (e.g., ion channels, neuropeptides, NT receptors), which prevents the characterization of Ebf2 function in a comprehensive manner. Future molecular profiling studies are needed to generate MMC-specific markers that will enable us to determine the degree of functional conservation between mouse Ebf2 and nematode UNC-3 in axial MN development. A potential outcome of such profiling studies is the identification of genes that are either expressed in all MMC neurons or in specific subsets of them, as suggested by our observation that ~ 40% of MMC neurons express *Ebf2*. The availability of such markers would not only decipher the extent of molecular diversity within the MMC neurons [1], but also aid current efforts for more precise molecular characterization of the identity of in vitro generated mammalian MNs, a much-anticipated goal in the field of in vitro modeling of MN disease [63–66]. Lastly, the identification of two novel markers (Ebf1, Ebf2) for post-mitotic axial MNs (HMC, MMC) brings us one step closer to reaching this goal.

Comparison of the role of COE family TFs in MNs of nematodes, simple chordates, and mice (this study) suggests that COE TFs played an ancient role in axial muscle-innervating MNs. In the nematode *C. elegans*, the sole COE ortholog UNC-3 is expressed in post-mitotic MNs that innervate axial muscle [21, 25]. This is also the case for the sole COE ortholog in the simple chordate *Ciona intestinalis* [21]. Interestingly, the zebrafish COE ortholog *Zcoe2* appears to also be expressed in axial muscle-innervating MNs [29]. Here, we describe that mEbf1 and mEbf2 are respectively expressed in HMC and MMC post-mitotic mouse MNs, which innervate distinct groups of axial muscle (HMC neurons > hypaxial muscle; MMC neurons > epaxial muscle, [Fig. 1c]). It has been suggested that HMC and MMC MNs likely reflect the vestige of an ancestral spinal motor column organization from which other motor columns derived in vertebrates [1, 2]. During this process, we propose that COE ortholog expression was maintained in axial muscle-innervating MNs (HMC and MMC), but lost in other motor columns, such as the limb-innervating MNs of the LMC. A speculative interpretation of our findings is that COE-type TFs may have controlled the specification of axial muscle-innervating MNs in the common ancestor of nematodes, simple chordates and vertebrates. We note, however, that the sole COE ortholog in flies *collier/knot* is not expressed in body wall-muscle innervating MNs [26]. Fly MNs use glutamate (Glu) as a neurotransmitter, whereas *C. elegans, Ciona intestinalis,* zebrafish and mouse MNs use acetylcholine (ACh). It is tempting to speculate that both cholinergic and glutamatergic MNs were present in the common ancestor to all bilaterians, which is thought to have had a fairly complex nervous system and likely used an axial-like locomotor circuit to move [1, 67, 68], but COE-type TFs were selectively expressed in the cholinergic MNs.

The function of axial muscle-innervating MNs is different between vertebrates and invertebrates [1]. For example, axial muscle-innervating MNs in *C. elegans* are required for locomotion, whereas, in mice, HMC and MMC neurons have adopted a more specialized role (HMC, breathing; MMC, maintenance of spinal alignment). To accommodate this functional change, it is conceivable that during evolution modifications have occurred in the molecular programs that specify axial muscle-innervating MNs. In *C. elegans* axial MNs, UNC-3 is required for axon guidance, ACh biosynthesis, expression of conserved MN-specific TFs (e.g., *ceh-12*/Hb9 or Mnx1), and induction of a large battery of MN identity-defining genes (e.g., ion channels, neuropeptides, NT receptors) [21, 25]. However, our analysis of mice lacking Ebf2 (UNC-3 ortholog) revealed a more specialized role for Ebf2 in axial muscle-innervating MNs. We did not observe axon guidance defects or reduction in expression of genes critical for ACh biosynthesis (e.g., VAChT, Acly) in MMC neurons of Ebf2 KO mice. Although Ebf2 is able to induce VAChT gene expression in *C. elegans* MNs (Fig. 4), this ability has been lost in mouse MMC neurons (Fig. 2a), which appear to have evolved a new genetic program orchestrated by the LIM homeodomain TF Islet1 for the control of ACh biosynthesis genes [69]. However, we found that – similar to *unc-3* mutant nematodes – the number of axial MNs expressing the conserved MN determinant Hb9 is significantly reduced in Ebf2 KO mice, indicating that aspects of the pre-existing, UNC-3-mediated genetic program present in *C. elegans* MNs have been conserved.

Our comparative study has evo-devo implications pertaining to the long-standing question of whether conservation of TF expression in orthologous cell types of different species is accompanied by conservation of TF function in these cell types. Through cross-species transgenic rescue experiments, we found that mEbf1 and mEbf2 can functionally substitute for *unc-3* function in *C. elegans* MNs, as assessed by monitoring expression of two MN identity markers (*acr-2*/AChR and *unc-17*/VAChT) (Fig. 4b-c). Quite remarkably, mEbf1 and mEbf2 were also able to partially rescue the severe locomotory defects observed in *unc-3* nematodes (Fig. 4d), indicating that expression of most, if not all, UNC-3 target genes is partly restored. These findings support the view that during evolution the structure and domain architecture of COE TFs did not significantly diverge. Indeed, there is high similarity between the DNA-binding and helix-loop-helix (HLH) domains of UNC-3, mEbf1 and mEbf2 [22, 29]. Moreover, a phylogenetic comparison among all 4 mouse Ebfs and UNC-3 revealed that mEbf1 and mEbf2 are more closely related to UNC-3 (Fig. 4a). We therefore surmise that the different functions of nematode UNC-3 and mouse Ebf2 in axial muscle-innervating MNs, i.e., UNC-3, but not Ebf2, controls ACh biosynthesis, may have arisen due to divergence at the level of their target genes (e.g., loss of unique sets of UNC-3 target genes in mouse MNs).

## Conclusions

Neuronal control of muscle associated with the central body axis is an ancient and essential function of both invertebrate and vertebrate nervous systems. Here, we provide evidence for an ancient role of the COE family of TFs in axial MN development. In the future, it will be interesting to examine whether other evolutionarily conserved TFs (e.g., *bnc-1*/BNC, *cfi-1*/Arid3a) that work together with UNC-3 to determine axial MN differentiation in *C. elegans* also control aspects of axial MN development in mice [19]. Further cross-species comparisons of the genetic programs underlying axial MN development may provide valuable insights into how axial MN diversity is generated and how distinct functions of axial MNs have evolved across the animal kingdom. Beyond MNs, our study highlights the usefulness of cross-species comparisons for the identification of genetic programs that orchestrate neuronal fate specification.

## Additional files

Additional file 1: **Figure S2.** Distribution analysis of Ebf2-expressing motor neurons along the medio-lateral axis of the MMC. A-B: All MMC nuclei were labeled with the Lhx3 marker (red nuclear signal) in Ebf2^lacZ/+^ spinal cords at e12.5 (*N* = 3). The position of Ebf2-expressing motor neurons (anti-bGal, green signal) within the MMC column was analyzed along the medio-lateral axis at three specific rostro-caudal regions of the spinal cord (rostral brachial, caudal brachial, thoracic). Below each image, arbitrary units (a. u.) of bGal fluorescence intensity are shown along the medio-lateral axis of the MMC (see Methods). C: Schematic summary of data shown in panel B. At rostral brachial and thoracic regions, Ebf2 positive cells are mainly located at the center of the MMC column, whereas at caudal brachial regions, Ebf2 motor neurons are mainly located medially within the MMC. (PDF 4639 kb)

Additional file 2: **Figure S1.** Additional characterization of Ebf expression in the mouse spinal cord. A: RNA ISH at e13.5 reveals Ebf1, Ebf2 and Ebf3 expression in DRG neurons (red circles). B: Double immunofluorescence staining for bGal (Ebf2 reporter in green) and Lhx3 (MMC marker in red) reveals co-localization (arrows) at a late embryonic stage (e18.5). A representative image is shown from the lumbar region. A subset of Lhx3 positive neurons expresses bGal (Ebf2), which is also the case at earlier (e13.5) stages (shown in Fig. 1e). However, we did not detect expression of Ebf2 in post-natal stages using either immunofluorescence or RNA ISH (data not shown). C: Antibody staining for the MN marker (Hb9, green signal) combined with fluorescent RNA ISH for *Ebf1* (red signal) revealed co-localization in WT e13.5 spinal cord selectively at thoracic levels, indicating *Ebf1* expression in HMC neurons. *N* = 3. (PDF 2267 kb)

## Abbreviations

HMC: Hypaxial motor column
KO mice: Knock-out mice
LMC: Lateral motor column
MMC: Medial motor column
MN: Motor neuron
TF: Transcription factor
WT: Wild type

## References

[1] D’Elia KP, Dasen JS (2018). Development, functional organization, and evolution of vertebrate axial motor circuits. Neural Dev.

[2] Dasen JS, Jessell TM (2009). Hox networks and the origins of motor neuron diversity. Curr Top Dev Biol..

[3] Philippidou P, Dasen JS (2013). Hox genes: choreographers in neural development, architects of circuit organization. Neuron..

[4] Dalla Torre di Sanguinetto SA, Dasen JS, Arber S (2008). Transcriptional mechanisms controlling motor neuron diversity and connectivity. Curr Opin Neurobiol..

[5] Stifani N (2014). Motor neurons and the generation of spinal motor neuron diversity. Front Cell Neurosci..

[6] Sharma K, Sheng HZ, Lettieri K, Li H, Karavanov A, Potter S (1998). LIM homeodomain factors Lhx3 and Lhx4 assign subtype identities for motor neurons. Cell..

[7] Catela C, Shin MM, Dasen JS (2015). Assembly and function of spinal circuits for motor control. Annu Rev. Cell Dev Biol..

[8] Dasen JS, De Camilli A, Wang B, Tucker PW, Jessell TM (2008). Hox repertoires for motor neuron diversity and connectivity gated by a single accessory factor, FoxP1. Cell..

[9] Hanley O, Zewdu R, Cohen LJ, Jung H, Lacombe J, Philippidou P (2016). Parallel Pbx-Dependent Pathways Govern the Coalescence and Fate of Motor Columns. Neuron..

[10] De Marco Garcia NV, Jessell TM (2008). Early motor neuron pool identity and muscle nerve trajectory defined by postmitotic restrictions in Nkx6.1 activity. Neuron..

[11] Fisher RE, Smith HF, Kusumi K, Tassone EE, Rawls A, Wilson-Rawls J (2012). Mutations in the Notch pathway alter the patterning of multifidus. Anat Rec (Hoboken)..

[12] Thor S, Thomas JB (2002). Motor neuron specification in worms, flies and mice: conserved and ‘lost’ mechanisms. Curr Opin Genet Dev..

[13] Doe CQ, Smouse D, Goodman CS (1988). Control of neuronal fate by the Drosophila segmentation gene even-skipped. Nature..

[14] Fujioka M, Lear BC, Landgraf M, Yusibova GL, Zhou J, Riley KM (2003). Even-skipped, acting as a repressor, regulates axonal projections in Drosophila. Development..

[15] Labrador JP, O’Keefe D, Yoshikawa S, McKinnon RD, Thomas JB, Bashaw GJ (2005). The homeobox transcription factor even-skipped regulates netrin-receptor expression to control dorsal motor-axon projections in Drosophila. Curr Biol..

[16] Landgraf M, Roy S, Prokop A, VijayRaghavan K, Bate M (1999). even-skipped determines the dorsal growth of motor axons in Drosophila. Neuron..

[17] Esmaeili B, Ross JM, Neades C, Miller DM, Ahringer J (2002). C. elegans even-skipped homologue, vab-7, specifies DB motoneurone identity and axon trajectory. Development..

[18] Moran-Rivard L, Kagawa T, Saueressig H, Gross MK, Burrill J, Goulding M (2001). Evx1 is a postmitotic determinant of v0 interneuron identity in the spinal cord. Neuron..

[19] Kerk SY, Kratsios P, Hart M, Mourao R, Hobert O (2017). Diversification of *C. elegans* Motor Neuron Identity via Selective Effector Gene Repression. Neuron..

[20] Kratsios P, Kerk SY, Catela C, Liang J, Vidal B, Bayer EA, et al. An intersectional gene regulatory strategy defines subclass diversity of *C. elegans* motor neurons. Elife. 2017;6. 10.7554/eLife.25751 PMC5498135 28677525

[21] Kratsios P, Stolfi A, Levine M, Hobert O (2012). Coordinated regulation of cholinergic motor neuron traits through a conserved terminal selector gene. Nat Neurosci..

[22] Pang K, Matus DQ, Martindale MQ (2004). The ancestral role of COE genes may have been in chemoreception: evidence from the development of the sea anemone, *Nematostella vectensis* (Phylum Cnidaria; Class Anthozoa). Dev Genes Evol..

[23] Kim K, Colosimo ME, Yeung H, Sengupta P (2005). The UNC-3 Olf/EBF protein represses alternate neuronal programs to specify chemosensory neuron identity. Dev Biol..

[24] Prasad B, Karakuzu O, Reed RR, Cameron S (2008). unc-3-dependent repression of specific motor neuron fates in *Caenorhabditis elegans*. Dev Biol..

[25] Prasad BC, Ye B, Zackhary R, Schrader K, Seydoux G, Reed RR (1998). unc-3, a gene required for axonal guidance in *Caenorhabditis elegans*, encodes a member of the O/E family of transcription factors. Development..

[26] Demilly A, Simionato E, Ohayon D, Kerner P, Garces A, Vervoort M (2011). Coe genes are expressed in differentiating neurons in the central nervous system of protostomes. PLoS One..

[27] Crozatier M, Vincent A (2008). Control of multidendritic neuron differentiation in Drosophila: the role of Collier. Dev Biol..

[28] Pozzoli O, Bosetti A, Croci L, Consalez GG, Vetter ML (2001). Xebf3 is a regulator of neuronal differentiation during primary neurogenesis in Xenopus. Dev Biol..

[29] Bally-Cuif L, Dubois L, Vincent A (1998). Molecular cloning of Zcoe2, the zebrafish homolog of Xenopus Xcoe2 and mouse EBF-2, and its expression during primary neurogenesis. Mech Dev..

[30] Chiara F, Badaloni A, Croci L, Yeh ML, Cariboni A, Hoerder-Suabedissen A (2012). Early B-cell factors 2 and 3 (EBF2/3) regulate early migration of Cajal-Retzius cells from the cortical hem. Dev Biol..

[31] Croci L, Chung SH, Masserdotti G, Gianola S, Bizzoca A, Gennarini G (2006). A key role for the HLH transcription factor EBF2COE2,O/E-3 in Purkinje neuron migration and cerebellar cortical topography. Development..

[32] Garel S, Garcia-Dominguez M, Charnay P (2000). Control of the migratory pathway of facial branchiomotor neurones. Development..

[33] Garel S, Marin F, Grosschedl R, Charnay P (1999). Ebf1 controls early cell differentiation in the embryonic striatum. Development..

[34] Garel S, Marin F, Mattei MG, Vesque C, Vincent A, Charnay P (1997). Family of Ebf/Olf-1-related genes potentially involved in neuronal differentiation and regional specification in the central nervous system. Dev Dyn..

[35] Wang MM, Reed RR (1993). Molecular cloning of the olfactory neuronal transcription factor Olf-1 by genetic selection in yeast. Nature..

[36] Wang SS, Lewcock JW, Feinstein P, Mombaerts P, Reed RR (2004). Genetic disruptions of O/E2 and O/E3 genes reveal involvement in olfactory receptor neuron projection. Development..

[37] Baumgardt M, Miguel-Aliaga I, Karlsson D, Ekman H, Thor S (2007). Specification of neuronal identities by feedforward combinatorial coding. PLoS Biol..

[38] Hattori Y, Sugimura K, Uemura T (2007). Selective expression of Knot/Collier, a transcriptional regulator of the EBF/Olf-1 family, endows the Drosophila sensory system with neuronal class-specific elaborated dendritic patterns. Genes Cells..

[39] Hattori Y, Usui T, Satoh D, Moriyama S, Shimono K, Itoh T (2013). Sensory-neuron subtype-specific transcriptional programs controlling dendrite morphogenesis: genome-wide analysis of Abrupt and Knot/Collier. Dev Cell..

[40] Jinushi-Nakao S, Arvind R, Amikura R, Kinameri E, Liu AW, Moore AW (2007). Knot/Collier and cut control different aspects of dendrite cytoskeleton and synergize to define final arbor shape. Neuron..

[41] Dubois L, Bally-Cuif L, Crozatier M, Moreau J, Paquereau L, Vincent A (1998). XCoe2, a transcription factor of the Col/Olf-1/EBF family involved in the specification of primary neurons in Xenopus. Curr Biol..

[42] Jin K, Jiang H, Mo Z, Xiang M (2010). Early B-cell factors are required for specifying multiple retinal cell types and subtypes from postmitotic precursors. J Neurosci..

[43] Corradi A, Croci L, Broccoli V, Zecchini S, Previtali S, Wurst W (2003). Hypogonadotropic hypogonadism and peripheral neuropathy in Ebf2-null mice. Development..

[44] Hoxha E, Tonini R, Montarolo F, Croci L, Consalez GG, Tempia F (2013). Motor dysfunction and cerebellar Purkinje cell firing impairment in Ebf2 null mice. Mol Cell Neurosci..

[45] Blackburn PR, Barnett SS, Zimmermann MT, Cousin MA, Kaiwar C, Pinto EVF (2017). Novel de novo variant in EBF3 is likely to impact DNA binding in a patient with a neurodevelopmental disorder and expanded phenotypes: patient report, in silico functional assessment, and review of published cases. Cold Spring Harb Mol Case Stud..

[46] Chao HT, Davids M, Burke E, Pappas JG, Rosenfeld JA, McCarty AJ (2017). A Syndromic Neurodevelopmental Disorder Caused by De Novo Variants in EBF3. Am J Hum Genet..

[47] Harms FL, Girisha KM, Hardigan AA, Kortum F, Shukla A, Alawi M (2017). Mutations in EBF3 Disturb Transcriptional Profiles and Cause Intellectual Disability, Ataxia, and Facial Dysmorphism. Am J Hum Genet..

[48] Klopocki E, Fiebig B, Robinson P, Tonnies H, Erdogan F, Ropers HH (2006). A novel 8 Mb interstitial deletion of chromosome 8p12-p21.2. Am J Med Genet A..

[49] Sleven H, Welsh SJ, Yu J, Churchill ME, Wright CF, Henderson A (2017). De Novo Mutations in EBF3 Cause a Neurodevelopmental Syndrome. Am J Hum Genet..

[50] Dasen JS, Tice BC, Brenner-Morton S, Jessell TM (2005). A Hox regulatory network establishes motor neuron pool identity and target-muscle connectivity. Cell..

[51] Schindelin J, Arganda-Carreras I, Frise E, Kaynig V, Longair M, Pietzsch T (2012). Fiji: an open-source platform for biological-image analysis. Nat Methods..

[52] Brenner S (1974). The genetics of *Caenorhabditis elegans*. Genetics..

[53] Nawa M, Kage-Nakadai E, Aiso S, Okamoto K, Mitani S, Matsuoka M (2012). Reduced expression of BTBD10, an Akt activator, leads to motor neuron death. Cell Death Differ..

[54] Chevenet F, Brun C, Banuls AL, Jacq B, Christen R (2006). TreeDyn: towards dynamic graphics and annotations for analyses of trees. BMC Bioinformatics..

[55] Dereeper A, Guignon V, Blanc G, Audic S, Buffet S, Chevenet F (2008). Phylogeny.fr: robust phylogenetic analysis for the non-specialist. Nucleic Acids Res.

[56] Guindon S, Dufayard JF, Lefort V, Anisimova M, Hordijk W, Gascuel O (2010). New algorithms and methods to estimate maximum-likelihood phylogenies: assessing the performance of PhyML 3.0. Syst Biol..

[57] Smith CL, Hollyday M (1983). The development and postnatal organization of motor nuclei in the rat thoracic spinal cord. J Comp Neurol..

[58] Alaynick WA, Jessell TM, Pfaff SL (2011). SnapShot: spinal cord development. Cell..

[59] Wichterle H, Lieberam I, Porter JA, Jessell TM (2002). Directed differentiation of embryonic stem cells into motor neurons. Cell..

[60] Bressenot A, Marchal S, Bezdetnaya L, Garrier J, Guillemin F, Plenat F (2009). Assessment of apoptosis by immunohistochemistry to active caspase-3, active caspase-7, or cleaved PARP in monolayer cells and spheroid and subcutaneous xenografts of human carcinoma. J Histochem Cytochem..

[61] Eiden LE (1998). The cholinergic gene locus. J Neurochem..

[62] Chung SH, Marzban H, Croci L, Consalez GG, Hawkes R (2008). Purkinje cell subtype specification in the cerebellar cortex: early B-cell factor 2 acts to repress the zebrin II-positive Purkinje cell phenotype. Neuroscience..

[63] Ho R, Sances S, Gowing G, Amoroso MW, O’Rourke JG, Sahabian A (2016). ALS disrupts spinal motor neuron maturation and aging pathways within gene co-expression networks. Nat Neurosci..

[64] Rhee HS, Closser M, Guo Y, Bashkirova EV, Tan GC, Gifford DK (2016). Expression of Terminal Effector Genes in Mammalian Neurons Is Maintained by a Dynamic Relay of Transient Enhancers. Neuron..

[65] Sances S, Bruijn LI, Chandran S, Eggan K, Ho R, Klim JR (2016). Modeling ALS with motor neurons derived from human induced pluripotent stem cells. Nat Neurosci..

[66] Velasco S, Ibrahim MM, Kakumanu A, Garipler G, Aydin B, Al-Sayegh MA (2017). A Multi-step Transcriptional and Chromatin State Cascade Underlies Motor Neuron Programming from Embryonic Stem Cells. Cell Stem Cell..

[67] Arendt D, Denes AS, Jekely G, Tessmar-Raible K (2008). The evolution of nervous system centralization. Philos Trans R Soc Lond B Biol Sci..

[68] De Robertis EM, Sasai Y (1996). A common plan for dorsoventral patterning in Bilateria. Nature..

[69] Cho HH, Cargnin F, Kim Y, Lee B, Kwon RJ, Nam H (2014). Isl1 directly controls a cholinergic neuronal identity in the developing forebrain and spinal cord by forming cell type-specific complexes. PLoS Genet..

