# Supplementary figures and images for: An ancient role for collier/Olf/Ebf (COE)-type transcription factors in axial motor neuron development
Source: Neural Dev. 2019 Jan 18;14:2. doi: 10.1186/s13064-018-0125-6 (PMC6339399; doi:10.1186/s13064-018-0125-6)

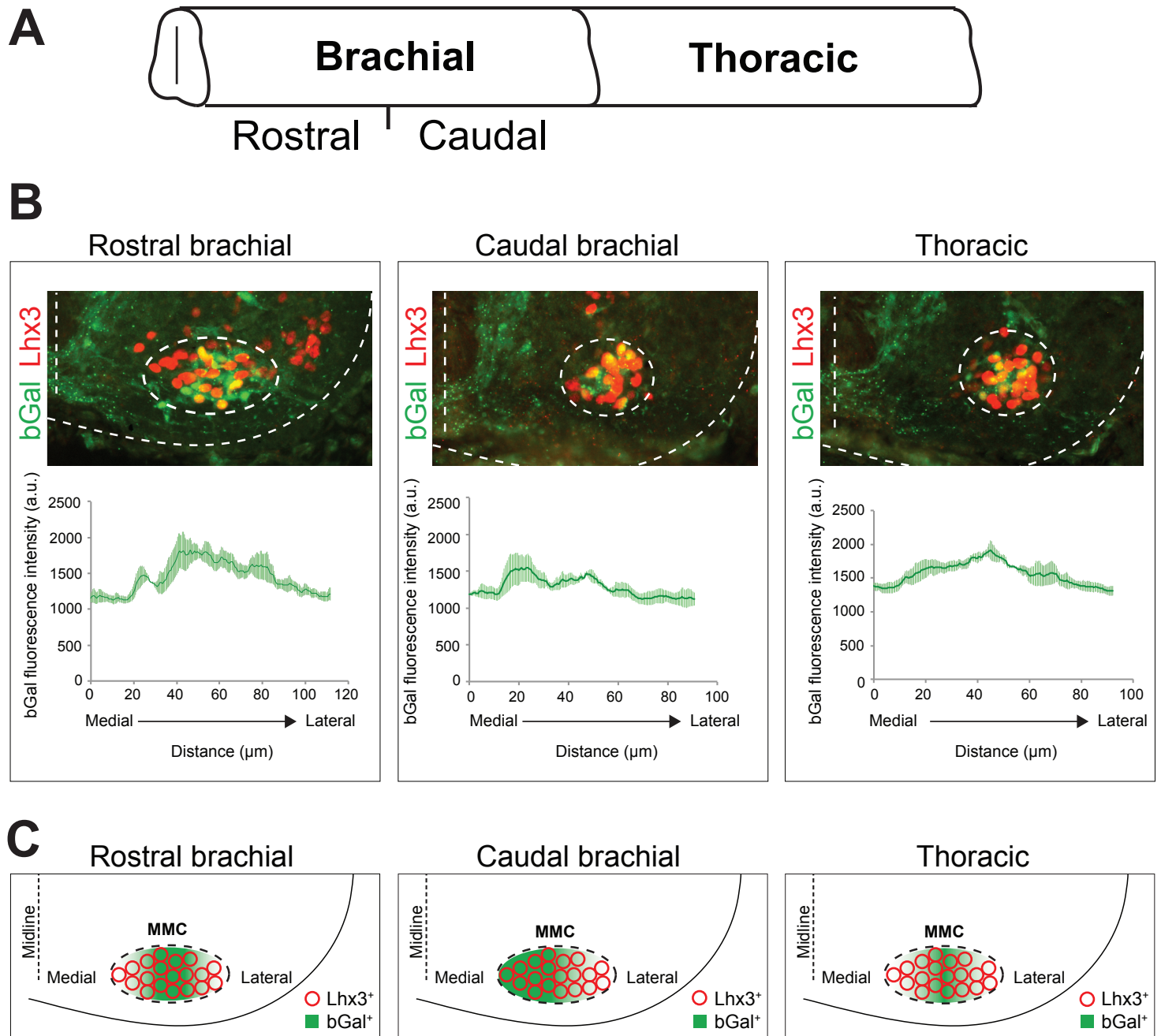

Supplement: Supplementary file 1 — Figure S2. Distribution analysis of Ebf2-expressing motor neurons along the medio-lateral axis of the MMC. A-B: All MMC nuclei were labeled with the Lhx3 marker (red nuclear signal) in Ebf2lacZ/+ spinal cords at e12.5 (N = 3). The position of Ebf2-expressing motor neurons (anti-bGal, green signal) within the MMC column was analyzed along the medio-lateral axis at three specific rostro-caudal regions of the spinal cord (rostral brachial, caudal brachial, thoracic). Below each image, arbitrary units (a. u.) of bGal fluorescence intensity are shown along the medio-lateral axis of the MMC (see Methods). C: Schematic summary of data shown in panel B. At rostral brachial and thoracic regions, Ebf2 positive cells are mainly located at the center of the MMC column, whereas at caudal brachial regions, Ebf2 motor neurons are mainly located medially within the MMC. (PDF 4639 kb) [file 13064_2018_125_MOESM1_ESM.pdf]

**A**

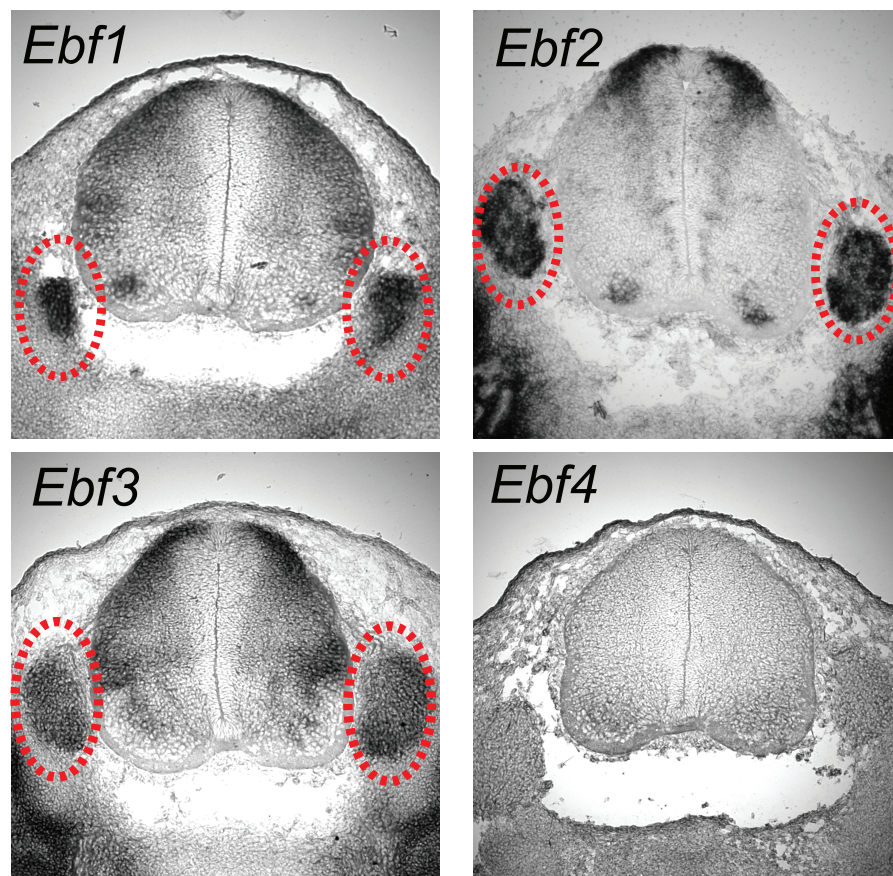

**B**

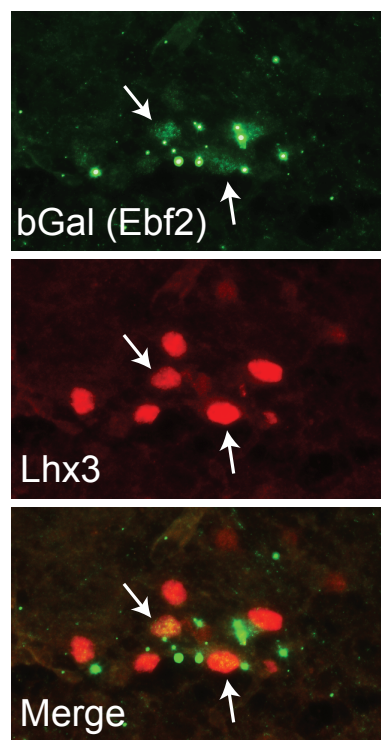

**C**

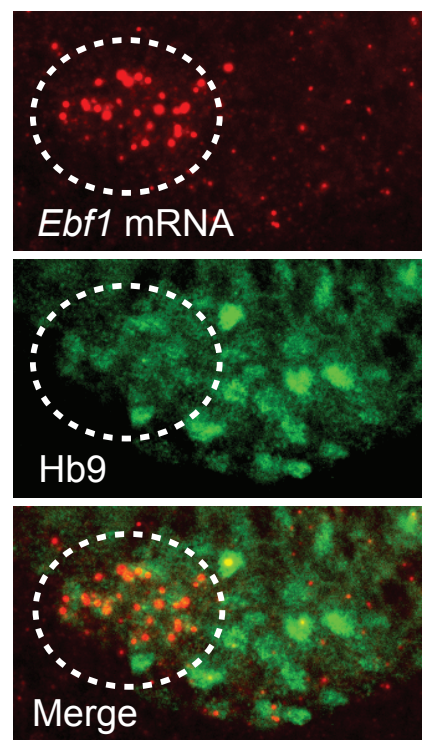

Supplement: Supplementary file 2 — Figure S1. Additional characterization of Ebf expression in the mouse spinal cord. A: RNA ISH at e13.5 reveals Ebf1, Ebf2 and Ebf3 expression in DRG neurons (red circles). B: Double immunofluorescence staining for bGal (Ebf2 reporter in green) and Lhx3 (MMC marker in red) reveals co-localization (arrows) at a late embryonic stage (e18.5). A representative image is shown from the lumbar region. A subset of Lhx3 positive neurons expresses bGal (Ebf2), which is also the case at earlier (e13.5) stages (shown in Fig. 1e). However, we did not detect expression of Ebf2 in post-natal stages using either immunofluorescence or RNA ISH (data not shown). C: Antibody staining for the MN marker (Hb9, green signal) combined with fluorescent RNA ISH for Ebf1 (red signal) revealed co-localization in WT e13.5 spinal cord selectively at thoracic levels, indicating Ebf1 expression in HMC neurons. N = 3. (PDF 2267 kb) [file 13064_2018_125_MOESM2_ESM.pdf]
